# Chemical Reduction of Nitrate by Zero-Valent Iron: Shrinking-Core versus Surface Kinetics Models

**DOI:** 10.3390/ijerph17041241

**Published:** 2020-02-14

**Authors:** Maria Villen-Guzman, Juan Manuel Paz-Garcia, Brahim Arhoun, Maria del Mar Cerrillo-Gonzalez, Jose Miguel Rodriguez-Maroto, Carlos Vereda-Alonso, Cesar Gomez-Lahoz

**Affiliations:** Department of Chemical Engineering, University of Malaga, 29071 Malaga, Spain; mvillen@uma.es (M.V.-G.); juanma.paz@uma.es (J.M.P.-G.); arhoun.b@uma.es (B.A.); mcerrillog@uma.es (M.d.M.C.-G.); maroto@uma.es (J.M.R.-M.); lahoz@uma.es (C.G.-L.)

**Keywords:** nitrate reduction, zero valent iron, permeable reactive barrier, shrinking-core model

## Abstract

Zero valent iron (ZVI) is being used in permeable reactive barriers (PRB) for the removal of oxidant contaminants, from nitrate to chlorinated organics. A sound design of these barriers requires a good understanding of kinetics. Here we present a study of the kinetics of nitrate reduction under relatively low values of pH, from 2 to 4.5. We use a particle size of 0.42 mm, which is within the recommended size for PRBs (0.2 mm to 2.0 mm). In order to avoid possible mass-transfer limitations, a well-stirred reactor coupled with a fluidized bed reactor was used. The experiments were performed at constant pH values using a pH controller that allows to accurately track the amount of acid added. Since the reduction of $H^{+}$ to $H_{2}$ by the oxidation of ZVI will always be present for these pH values, blank experiments (without nitrate) were performed and the rate of this $H^{+}$ reduction obtained. This rate of reduction was studied using three kinetic models: a regular empirical one, the Shrinking-Core Model (SCM), and the Surface Kinetics Model (SKM). The best performance was obtained from the SKM model. Therefore, this model was also used to study the results for the nitrate reduction, also with satisfactory results. In both cases, some assumptions are introduced to maintain a moderate number of fitting parameters.

## 1. Introduction

The first pilot test of a permeable reactive barrier (PRB) for the in situ groundwater remediation of chlorinated organic compounds was performed at the Canadian Forces Base Borden in Ontario, Canada [1,2,3]. In that case, the permeable wall consisted of a mixture of granular iron and sand. Since then, PRBs have been used for the removal of chromium and other metals, as well as radionuclides and nitrates, using different materials as reactive media [4]. Today, PRB is recognized as a fully developed conventional technology.

Zero valent iron (ZVI) was demonstrated to be an effective reactive material for the removal of nitrate from contaminated groundwater by chemical reduction. A recent review of this topic [5] indicates that the physical and chemical characteristics of ZVI, as well as its operational conditions, affect not only the efficiency of the nitrate reduction, but also the distribution of the different end products. The operational variable that most significantly affects the rate of nitrate reduction is the pH value of the aqueous solution. Protons not only participate directly in the reduction of nitrates, but also affect the competitive parallel reactions such as iron corrosion. One of the main drawbacks of the nitrate reduction by ZVI is that the end product is mainly ammonium, which could be also a threat to the environment. More and more interest is focused on increasing the selectivity of nitrate reduction towards $N_{2}$ [5].

However, when nitrate is not the target pollutant, its reduction by ZVI to ammonium still occurs [6], and a competition between the target contaminant (i.e., chlorinated solvent, chromium, etc.) and nitrate will take place. These competitive reductions will affect the performance of the remediation process and should be considered in a sound PRB design. 

Different processes take place during the nitrate and co-oxidants reduction: diffusion from the aqueous solution towards the iron particle, adsorption onto the particle surface, chemical redox reaction, desorption of the reaction products and diffusion of those products into the aqueous solutions. An accurate description of all these processes taking place under natural conditions is rather difficult, and is far from being achieved today. A good engineering work will need to know the relative kinetics of all the processes taking place in series and in parallel, and pay attention only to those controlling the kinetics of the overall process. To do this, first of all, one should be able to know the chemical reaction kinetics under conditions that allow the best possible mass-transfer rates, so that these mass-transfer processes are not limiting the chemical reaction kinetics. The most important variables for the process are the relative surface of the solid particles and the pH value of the aqueous solutions [7,8,9,10,11]. The particle size recommended for ZVI PRBs is between 0.2 and 2 mm [9,12]. In this work we use particles within this interval (0.42 mm) and mildly acidic pH values. For these pH values the chemical reaction is rather fast, and it is more likely to be taking place under mass-transfer limitations. Obviously, to find out if these limitations are taking place, one should know the kinetics of the unlimited process first.

Therefore, the development of chemical-reaction kinetic models that are able to predict and describe the kinetics of nitrate reduction to ammonium is in any case necessary. As with any mathematical model, it should be kept as simple as possible while being able to describe the competitive effect between the different species in the reactive and/or adsorption processes.

In this study, the kinetics of the nitrate reduction to ammonium by granular ZVI in aqueous solution is examined. We use a fluidized bed reactor to maximize the mass-transfer kinetics so that we are sure that we are working under optimal conditions for the reaction process to take place under chemical reaction control. In addition to the nitrate reduction experiments, several control tests without nitrate were also performed in order to assess the importance of the competitive reduction of protons. Both types of experiments were performed at different controlled pH values with a constant ZVI dosage and at a constant temperature. Finally, different simple kinetics models were tested to describe: firstly, the corrosion of iron particles in the acid solution (proton reduction), and secondly, the reduction of nitrate by ZVI in an aqueous solution.

## 2. Materials and Methods

**Figure 1:**
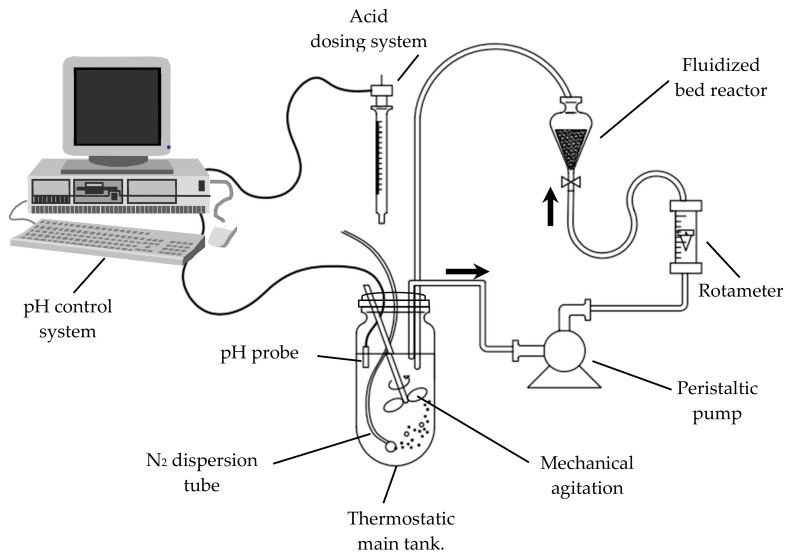
Schematic representation of the experimental system.

The experimental system (Figure 1) basically consists of a glass stirred tank reactor with a working volume of 5 L. The liquid phase in the tank was continuously pumped through a fluidized bed reactor, which contains the solid particles, and then returned to the main tank. As indicated above, this optimizes the contact between the solid phase (iron particles) and the liquid phase, with or without ${\mathrm{NO}}_{3}^{-}$. Thus, the behavior of the whole reaction system could be considered close enough to an ideal batch stirred tank reactor.

In the main tank:

- A Heidolph RZR1 (Heidolph Instruments GmbH & CO. KG, Schwabach, Germany) motor connected to a stirring shaft with a two-blade propeller provides mechanical agitation $\left(400\;\mathrm{rpm}\right)$;
- The dissolved oxygen concentration was maintained at zero by means of a continuous nitrogen current bubbled through the aqueous phase;
- A pH control system of our own design [8] allowed us to maintain the pH value within ±0.05 the target value, by the addition of the necessary volume of sulfuric acid solution. This device also made it possible to track the amount of acid added versus time.

The reactions were carried out isothermally, immersing the stirred tank in a thermostatic bath maintained at 15±1 °C. A peristaltic pump (Dinko Instruments, model D-25V (Dinko Instruments, Barcelona, Spain)) withdrew the solution from the main tank at a flow rate of $2.2\;{L\;\min}^{-1}$ and fed it to the bottom of the fluidized bed reactor. The latter consists of a conical-shaped vessel of 250 mL with the largest section at the highest position. The upward aqueous flow raises the iron particles of the bed located at the narrow section of the reactor and spreads them throughout the vessel. As the reactor section increases, the fluid velocity decreases, and with it the drag force, allowing the solid particles to fall.

A 5 L solution of $500\;\mathrm{mg}\;{\mathrm{NaNO}}_{3}\;L^{-1}$ was prepared with milli-Q water and with the required amount of sulfuric acid to reach the target pH value. After the solution was placed into the main tank of the reaction system, the mechanical agitation, nitrogen bubbling and pH control were started. Once the tank reached the required temperature, the pump was activated for 10 min and the solution circulated through the silicone tubes and the empty fluidized bed reactor, thus allowing all the system elements to reach 15 °C. Then, the pump was stopped and 60 g of iron particles (0.42 mm in diameter, Panreac, PRS) were loaded into the fluidized bed reactor. The test was started shortly afterwards, when pumping was resumed. Samples of 10 mL were withdrawn from the main tank at selected times and immediately stored in plastic vials at 4 °C until their analysis. The volume of liquid withdrawn in a test (12–15 samples) was partially compensated by the volume of sulfuric acid solution added by the pH control system; therefore, the volume changes in a test can be assumed negligible (<3%).

The aqueous concentrations of ammonium and nitrate were determined by ion chromatography (Hewlett Pacard 1050 series) with a 732 IC Detector (Metrohm). The IC-Pak Cation M/D Column (Waters) was used for ammonium determination using the mobile phase specified by the column manufacturer (0.0292 g of EDTA + 189 μL of 65% nitric acid + milli-Q water to get 1 L) at a flow rate of $1\;{\mathrm{mL}\;\min}^{-1}$. The Anion Metrosep column (Metrohm) was used for nitrate determination, with the mobile phase recommended by the column manufacturer (1.33 g of phthalic acid + 0.935 g of TRIS + 20 mL of acetonitrile + milli-Q water to get 1 L) at a flow rate of $0.8\;{\mathrm{mL}\;\min}^{-1}$. The aqueous iron concentration was determined by flame atomic absorption spectroscopy (Varian AA210 (Varian Inc., California, EE.UU.)).

Duplicated tests were performed at pH values of 2.0, 2.5, 3.5, and 4.5 to study the effect of the pH on the kinetics of nitrate reduction. In addition, duplicated control tests were also performed at the same pH values to study the competitive reduction of protons. The experimental procedure of these control tests was the same as described above, but without sodium nitrate.

## 3. Results and Discussion

### 3.1. Kinetics of the Corrosion of Iron in Acid

First, it is checked that the experimental results follow the expected stoichiometry for proton reduction in the control tests, as obtained from the rate of acid added by the pH controller. Then, 3 models are proposed to describe the kinetics of that reduction.

#### 3.1.1. Stoichiometry

**Figure 2:**
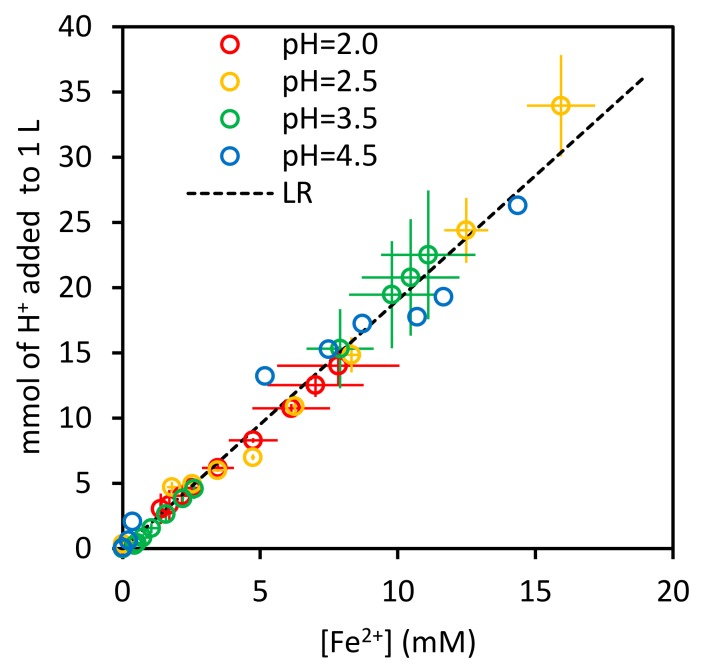
Stoichiometry of the Fe^0^ oxidation.

Figure 2 shows the consumption of protons required to keep a constant value of pH as a function of the concentration of Fe^2+^ in the aqueous phase for the 4 control tests. The slope of the linear regression (LR) of the proton consumption on the concentration of Fe^2+^ is 1.90±0.03 ($R^{2}=0.991$), a value close enough to that expected according to stoichiometry of the reaction (1).

(1) $$2\;H^{+}\left(aq\right)+{\mathrm{Fe}}^{0}\left(s\right)\to H_{2}\left(g\right)+{\mathrm{Fe}}^{2+}\left(aq\right)$$

#### 3.1.2. Empirical Rate Equation of *n*th Order

The experimental results indicate that iron corrodes in sulfuric acid at an almost constant rate, the rate being different for each pH tested. Hence, a simple empirical kinetics equation of *n*th order with respect to protons was tested.

(2) $$\frac{d\left[{\mathrm{Fe}}^{2+}\right]}{dt}=k\;{\left[H^{+}\right]}^{n}=slope\;\;∴\;\;\log\left(slope\right)=\log k-n\;pH$$

**Figure 3:**
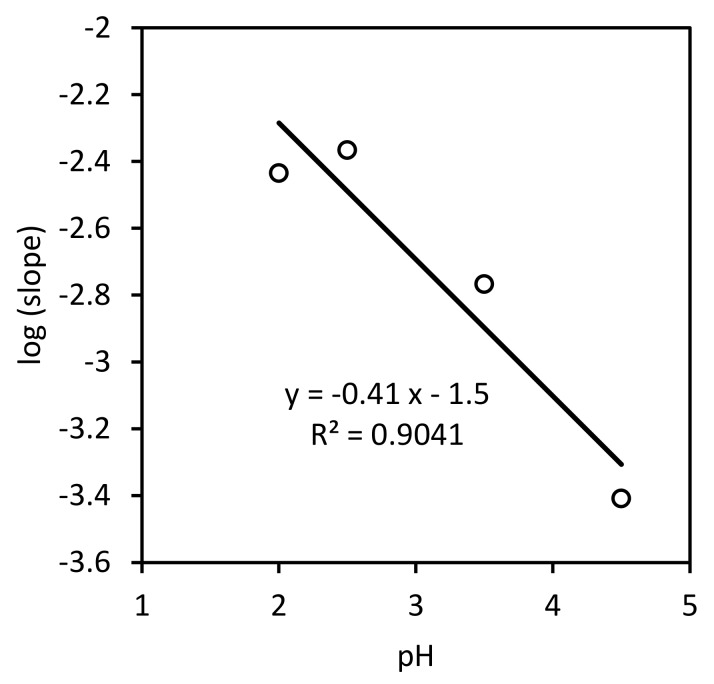
Slope values as a function of pH.

Even though at each constant pH value tested, the $\left[{\mathrm{Fe}}^{2+}\right]$-time experimental data lie on a straight line with zero intercept (not shown), the fit of the slope values calculated at each pH to the linearized expression as a function of pH is not good enough (Figure 3). Despite this, the calculated values for this model would be: $k=\left(0.03\pm 0.02\right)\;h^{-1}{\left({\mathrm{mol}\;L}^{-1}\right)}^{\left(1-0.41\right)}$ and n=0.41±0.09.

#### 3.1.3. Shrinking Unreacted-Core Model (SCM)

The SCM is a simple model frequently used for the non-catalytic reaction of particles with a surrounding fluid [13]. The model considers that the reacting particles behave as spheres that shrink during the reaction, finally disappearing. When the chemical reaction on the surface of the particles controls the overall process, the rate would be proportional to the surface of the unreacted core.

(3) $$-\frac{1}{4\pi r^{2}}\frac{dN_{\mathrm{Fe}}}{dt}=-\frac{1}{4\pi r^{2}}\;\frac{1}{2}\;\frac{dN_{H^{+}}}{dt}=\frac{1}{2}k_{s}{\left[H^{+}\right]}^{n}$$

where r is the radius of the unreacted core, $N_{i}$ is the number of moles of the component i, and $k_{s}$ is the rate constant for the surface reaction, which is assumed to be of *n*th order with respect to proton. That rate expression is written in terms of the shrinking radius of the unreacted core, using the molar density of the iron spheres $\left(\rho_{\mathrm{Fe}}\right)$.

(4) $$-\frac{1}{4\pi r^{2}}\;\rho_{\mathrm{Fe}}\;4\pi r^{2}\frac{dr}{dt}=-\rho_{\mathrm{Fe}}\;\frac{dr}{dt}=\frac{1}{2}k_{s}{\left[H^{+}\right]}^{n}$$

The integration of this equation allows the calculation of the core to shrink with time.

(5) $$t=\frac{\rho_{\mathrm{Fe}}}{\frac{1}{2}k_{s}{\left[H^{+}\right]}^{n}}\left(R-r\right)$$

where R is the radius of the spheres at t=0. If τ is the time required for a complete conversion of a core $\left(r=0\right)$, Equation (5) can be written as

(6) $$\tau =\frac{\rho_{\mathrm{Fe}}\;R}{\frac{1}{2}k_{s}{\left[H^{+}\right]}^{n}}\;\;∴\;\;\frac{t}{\tau }=1-\frac{r}{R}$$

Finally, the progression of reaction in terms of iron fractional conversion is obtained using the relationship between the iron fractional conversion $\left(X_{\mathrm{Fe}}\right)$ and the fraction of unreacted volume of a sphere.

(7) $$1-X_{\mathrm{Fe}}=\frac{\frac{4}{3}\pi r^{3}}{\frac{4}{3}\pi R^{3}}={\left(\frac{r}{R}\right)}^{3}\;\;∴\;\;\frac{t}{\tau }=1-{\left(1-X_{\mathrm{Fe}}\right)}^{1/3}$$

**Figure 4:**
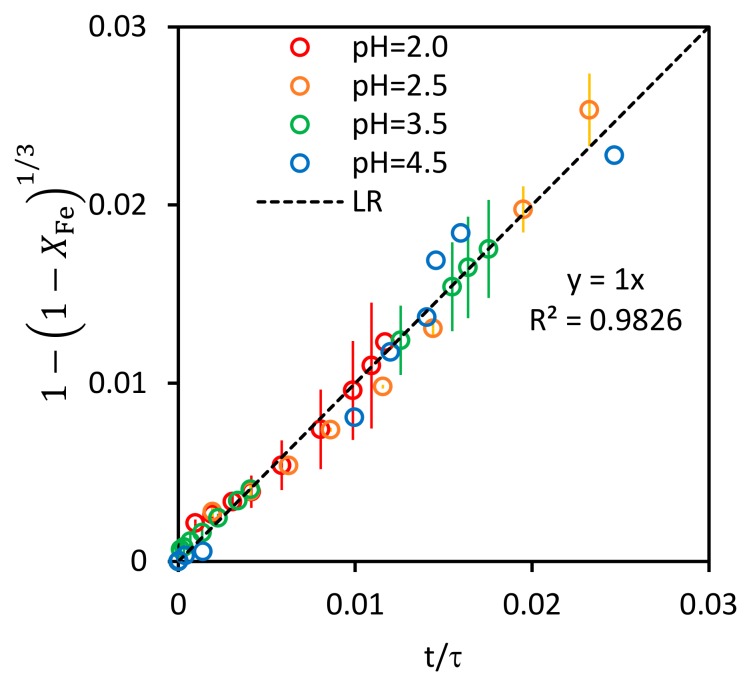
Progression of the reaction in terms of fractional time for complete conversion.

The values of τ for each pH tested are obtained by the fit of Equation (7) to the experimental values of iron fractional conversion (not shown). The values of the correlation coefficients for the 4 lineal fits are within the interval $\left[0.9875,\;0.9991\right]$. Figure 4 shows the experimental results of the four control tests using the fractional time for complete conversion as the x-axis. As expected, the slope of the linear regression of all those experimental data is 1.

Once the values of τ for each pH are known, the values of the rate constant and of the reaction order with respect to protons are obtained by the linear regression of $\log\left(\tau \right)$ on pH according to the linearization of Equation (6), which is shown as Equation (8).

(8) $$\log\left(\tau \right)=\log\left(\frac{2\;\rho_{\mathrm{Fe}}\;R}{k_{s}}\right)+n\;pH$$

**Figure 5:**
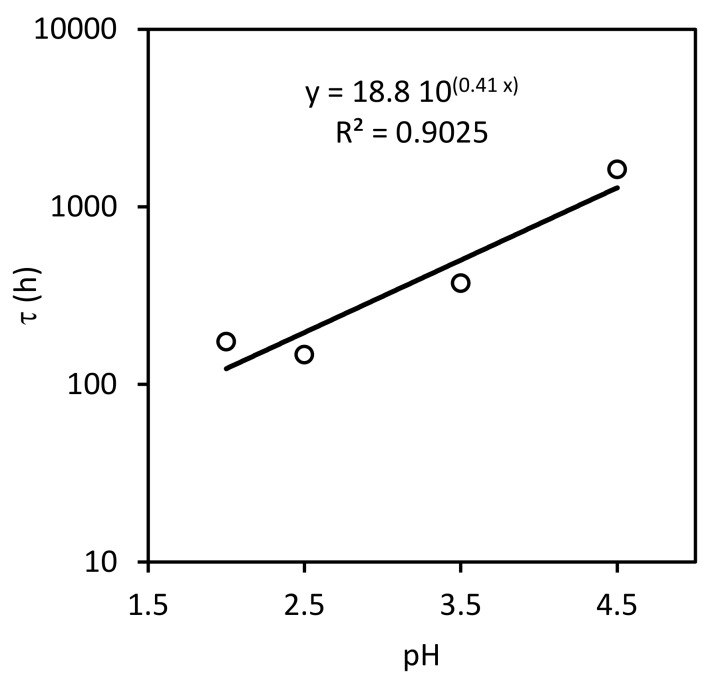
Time required for a complete conversion of a core (τ) as a function of pH.

This fit is shown in Figure 5, and the calculated values for the SCM parameters are: $k_{s}=\left(0.006\pm 0.004\right)\;{m\;h}^{-1}{\left({\mathrm{mol}\;L}^{-1}\right)}^{\left(1-0.41\right)}$ and n=0.41±0.09, using a particle diameter of 0.42 mm and an iron molar density of $141\;{\mathrm{mol}\;L}^{-1}$.

#### 3.1.4. Surface Kinetics Model (SKM)

This model assumes that the reaction takes place on active sites on the surface of the iron particles. If mass-transfer rates are fast enough, three steps should occur successively: adsorption of the reactants on the active sites, reaction either between adjacent adsorbed reactants (dual-site mechanism) or between adsorbed reactants and components in the fluid phase (single-site mechanism), and desorption of the products from the surface.

**Figure 6:**
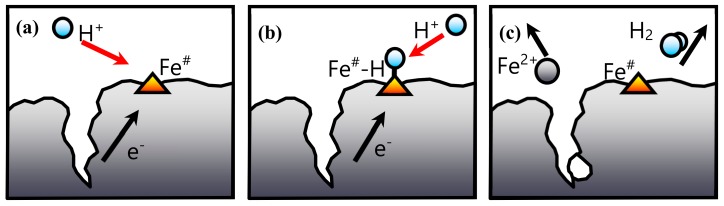
Superficial reaction steps: (**a**) adsorption; (**b**) reaction; (**c**) desorption.

In the acid corrosion of a metal surface, two different zones develop in the solid: a cathodic and an anodic zone [14]. In the present case, the reduction occurs at the cathodic zone, where a proton adsorbed on the iron surface is reduced (Figure 6). Those adsorbed protons will react between them, or with other protons in the fluid phase, to form the hydrogen gas. On the other hand, iron oxidation takes place in the anodic zone, where ${\mathrm{Fe}}^{2+}$ ions will be released into the aqueous phase. The high electrical conductivity of the metal allows an efficient transfer of electrons between the anodic zone and the cathodic zone.

If the surface of the cathodic zone is considered constant while the cracks in the anodic zone increase, the surface kinetics model could be used according to the following steps:

(1) Proton adsorption on the active site $\left({\mathrm{Fe}}^{\#}\right)$

(9) $$H^{+}+\left({\mathrm{Fe}}^{\#}\right)+\frac{1}{2}{\left(\mathrm{Fe}\right)}_{anodic}\;\begin{array}{c} \overset{\;\;\;\;\;k_{1}\;\;\;\;\;\;}{\to }\\ \underset{\;\;\;\;\;k_{-1}\;\;\;}{\leftarrow } \end{array}\;{\left({\mathrm{Fe}}^{\#}\cdot H\right)}_{ads}+\frac{1}{2}\;{\mathrm{Fe}}^{2+}$$

We consider that this adsorption is very fast as compared with the following reaction step, and therefore a dynamic adsorption–desorption equilibrium would be reached.

(2) Reduction of adsorbed protons, considering irreversible reaction with fast desorption of the reaction products

(10) $${\left({\mathrm{Fe}}^{\#}\cdot H\right)}_{ads}+H^{+}+\frac{1}{2}{\left(\mathrm{Fe}\right)}_{anodic}\;\overset{\;\;\;\;\;k_{2}\;\;\;\;\;\;}{\to }\;\left({\mathrm{Fe}}^{\#}\right)+\frac{1}{2}\;{\mathrm{Fe}}^{2+}+H_{2}\left(g\right)$$

where $H^{+}$ represents the second proton involved in the reaction (dissolved in the aqueous phase or adsorbed in the solid surface). In a first approach it could be assumed that the rate of this step is *n*th order with respect to protons and first order with respect to the fraction of the active site covered by protons, ${\left({\mathrm{Fe}}^{\#}\cdot H\right)}_{ads}$, which will be written as $\theta_{SH}$. Nevertheless, in order to maintain a number of fitting parameters equal to the previous models we have assumed that n=0. As will be seen later, the results obtained with this assumption are good enough. Thus, the rate equation for this SKM is expressed by:

(11) $$\frac{d\left[{\mathrm{Fe}}^{2+}\right]}{dt}=k_{2}\;\theta_{SH}$$

The fraction of the active site covered by protons can be expressed as a function of its aqueous concentration and the adsorption equilibrium constant $\left(K_{1}\right)$ corresponding to the Equation (9).

(12) $$K_{1}=\frac{k_{1}}{k_{-1}}=\frac{\theta_{SH}}{\theta_{S}\;\left[H^{+}\right]}\;\;∴\;\;\theta_{SH}=\frac{K_{1}\;\left[H^{+}\right]}{\;1+K_{1}\left[H^{+}\right]\;}$$

where $\theta_{S}$ is the fraction of active site uncovered. Note that $\theta_{S}+\theta_{SH}=1$. Equations (11) and (12) give the rate of reaction in terms of the proton aqueous concentration and only two fitting parameters.

(13) $$\frac{d\left[{\mathrm{Fe}}^{2+}\right]}{dt}=k_{2}\;\frac{K_{1}\;\left[H^{+}\right]}{\;1+K_{1}\left[H^{+}\right]\;}=m$$

Therefore, the rate of reaction should be constant in each control test, since the pH is maintained at a constant value. Those rates are obtained from the slopes $\left(m\right)$ of the linear regressions with zero intercept of $\left[{\mathrm{Fe}}^{2+}\right]$ vs. time experimental data (regressions not shown).

The values of $K_{1}$ and $k_{2}$ can be calculated from the linearized form of the Equation (13):

(14) $$\frac{1}{m}=\frac{1}{k_{2}\;K_{1}}\frac{1}{\left[H^{+}\right]}+\frac{1}{k_{2}}$$

**Figure 7:**
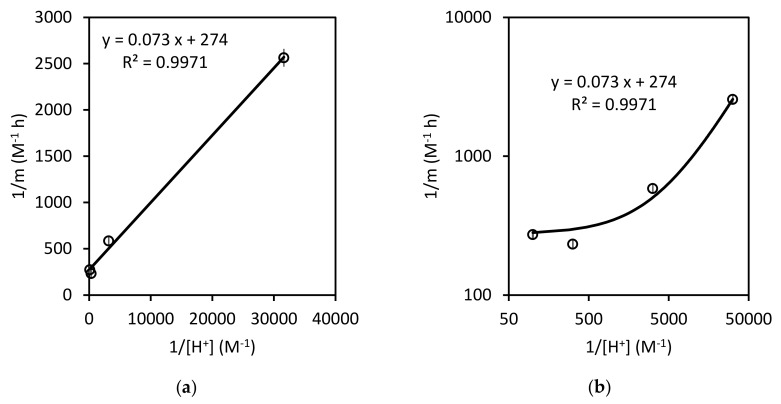
1/m values as a function of the inverse of proton concentration: (**a**) natural scale; (**b**) double-logarithmic scale.

As can be seen from the value of $R^{2}$ in Figure 7a, this fit is slightly better than the equivalent ones in the two models discussed previously. This value of $R^{2}$ may be overrated due to the accumulation of experimental values on the left-hand side of the figure. Nevertheless, Figure 7b shows the same results while using a double-logarithmic scale, so that the good performance of the model can be seen more clearly. The calculated values for the SKM parameters are: $\begin{array}{c} k_{2}=\left(0.0036\pm 0.0006\right)\;{\mathrm{mol}\;L}^{-1}{\;h}^{-1} \end{array}$ and $\begin{array}{c} K_{1}=\left(3\;800\pm 800\right)\;{\mathrm{mol}}^{-1}\;L \end{array}$.

**Figure 8:**
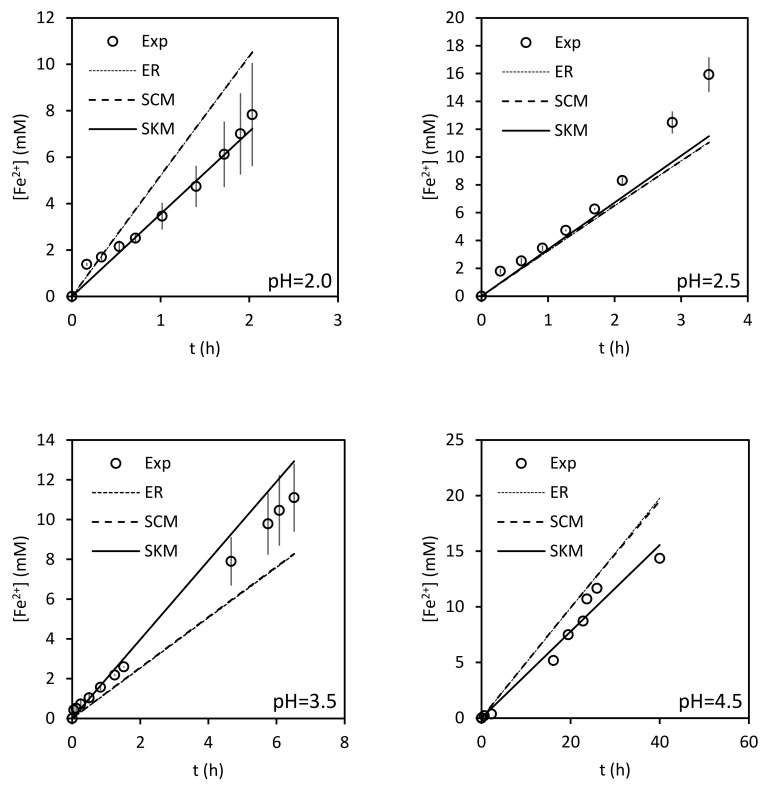
Experimental results and predictions of the 3 models.

The experimental results and the predictions of the three models are shown in Figure 8. As can be seen, the predictions obtained with the empirical rate equation of the *n*th order are indistinguishable from those of the SCM. Besides, even when the three models allow a similar prediction quality, the best performance is obtained for the SKM model, especially for the more acidic conditions. Obviously, an SKM without our simplifications would provide a better fit, but with the drawback of requiring additional parameters.

### 3.2. Kinetics of Nitrate Reduction

#### 3.2.1. Stoichiometry

The stoichiometry of nitrate reduction to ammonia by the oxidation of ZVI to Fe^2+^ is given by:

(15) $${\mathrm{NO}}_{3}^{-}\left(aq\right)+4\;{\mathrm{Fe}}^{0}\left(s\right)+10\;H^{+}\left(aq\right)\to {\mathrm{NH}}_{4}^{+}\left(aq\right)+\;4\;{\mathrm{Fe}}^{2+}\left(aq\right)+3\;H_{2}O$$

For this reaction the ratios of ${\mathrm{NO}}_{3}^{-}$ consumption to ${\mathrm{Fe}}^{2+}$ production and to $H^{+}$ consumption would be 0.25 and 0.1, respectively, and therefore the ratio of $H^{+}$ consumption to ${\mathrm{Fe}}^{2+}$ production would be 2.5. For the parallel reaction (Equation (1) discussed above) where $H^{+}$ is reduced to $H_{2}$, the ratio of $H^{+}$ consumption to ${\mathrm{Fe}}^{2+}$ produced would be 2. 

**Figure 9:**
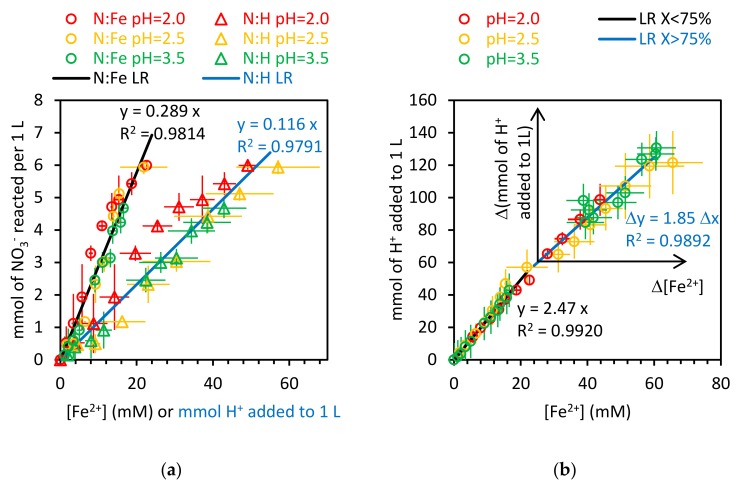
Stoichiometry of the reactions studied: (**a**) ${\mathrm{NO}}_{3}^{-}:{\mathrm{Fe}}^{2+}$ and ${\mathrm{NO}}_{3}^{-}:H^{+}$ ratios for nitrate conversions below 75%; (**b**) $H^{+}:{\mathrm{Fe}}^{2+}$ ratios for nitrate conversions below and above 75%.

Figure 9a shows the experimental values of nitrate concentration depletion against Fe^2+^ concentration and also against the moles of $H^{+}$ added by the pH controller per liter of reactor. The solid lines for Figure 9a are the linear regressions with zero intercept for each set of values. As can be seen, the slope of these regression lines (0.289 and 0.116) are not far from the theoretical values that would be obtained if Equation (15) was the only reaction taking place (0.250 and 0.100).

Figure 9b shows the moles of $H^{+}$ added per liter and the Fe^2+^ concentration. Here there are two solid lines: the first one is the linear regression with zero intercept for the experimental values obtained for experiments where the nitrate concentration is above 25% of the initial one. The second one is also the linear regression with zero intercept, but using a new axis placed at the point where 75% of the initial nitrate is already converted. As can be seen, for the higher values of nitrate concentration, the slope of the linear regression is 2.47, again very close to the theoretical value, 2.5, that would be obtained if the reaction shown in Equation (15) were the only one taking place. For the lower values of nitrate concentration, the slope obtained for the linear regression is 1.85, much closer to the value that would be obtained if the oxidation of iron is produced by the reduction of $H^{+}$ (following Equation (1)).

Figure 9 does not include the experimental results obtained at a constant pH of 4.5, because the molar ratios calculated for the experiments at that pH are all significantly below the ratios shown in Figure 9. Additional reactions would probably have to be considered at pH 4.5, such as the formation of iron hydroxide. Therefore, the experiments at pH 4.5 will not be considered throughout the following discussion.

#### 3.2.2. Surface Kinetics Model (SKM)

When a simple empirical kinetic equation is fitted to the experimental data for nitrate concentration depletion vs. time, the order of reaction obtained with respect to nitrate is close to zero (*n* = 0.016) whereas the calculated order with respect to protons is 0.88 (not shown). Although this empirical rate equation can describe well enough the progress of nitrate reduction, it cannot simulate the shift in the reaction order with respect to nitrate that is usually observed as the concentration of nitrates decreases. However, the SKM is able to reproduce that kind of behavior. This fact, together with the slightly better reliability of the SKM for the parallel reaction discussed above, which involves proton reduction, indicates that this kind of model is a good option for the representation of both reactions.

In this case, as was explained above (see Figure 6), the model assumes a negative charge in the external surface (cathodic region) of the iron particle, where cations can be adsorbed. The adsorption of anions can only take place onto a position already covered by a cation. Similar assumptions have been published previously [15]. Thus, the model proposes the adsorption of nitrates onto the active site of the iron surface already covered by protons.

(16) $${\mathrm{NO}}_{3}^{-}+{\left({\mathrm{Fe}}^{\#}\cdot H\right)}_{ads}\;\begin{array}{c} \overset{\;\;\;\;\;k_{3}\;\;\;\;\;\;}{\to }\\ \underset{\;\;\;\;\;k_{-3}\;\;\;}{\leftarrow } \end{array}\;{\left({\mathrm{Fe}}^{\#}\cdot H\cdot {\mathrm{NO}}_{3}^{-}\right)}_{ads}$$

Again, this adsorption step is considered fast enough to allow dynamic adsorption–desorption equilibrium to be achieved. Therefore, the fraction of the active site covered by nitrates $\left(\theta_{SHN}\right)$ and by protons $\left(\theta_{SH}\right)$ can be expressed as a function of their aqueous concentration and the adsorption equilibrium constants $\left(K_{3},\;K_{1}\right)$. Note that now $\theta_{S}+\theta_{SH}+\theta_{SHN}=1.$

(17) $$\theta_{SH}=\frac{K_{1}\left[H^{+}\right]}{\;1+K_{1}\left[H^{+}\right]+K_{1}\;K_{3}\;\left[H^{+}\right]\;\left[{\mathrm{NO}}_{3}^{-}\right]\;}\;\;∴\;\;\theta_{SHN}=\frac{K_{1}\;K_{3}\;\left[H^{+}\right]\;\left[{\mathrm{NO}}_{3}^{-}\right]}{\;1+K_{1}\left[H^{+}\right]+K_{1}\;K_{3}\;\left[H^{+}\right]\;\left[{\mathrm{NO}}_{3}^{-}\right]\;}$$

The reduction of the adsorbed nitrate, which proceeds in parallel to the proton reduction (Equation (10)), is also considered an irreversible reaction with fast desorption of the reaction products.

(18) $${\left({\mathrm{Fe}}^{\#}\cdot H\cdot {\mathrm{NO}}_{3}^{-}\right)}_{ads}+9\;H^{+}+\frac{7}{2}\;{\left(\mathrm{Fe}\right)}_{anodic}\;\overset{\;\;\;\;\;k_{4}\;\;\;\;\;\;}{\to }\;{\mathrm{NH}}_{4}^{+}+\left({\mathrm{Fe}}^{\#}\right)+\frac{7}{2}\;{\mathrm{Fe}}^{2+}+3\;H_{2}O$$

As expected, the stoichiometry of the nitrate reduction as given by Equation (15) agrees with the sum of Equations (9), (16), and (18), that represent the overall mechanism of the SKM model.

Again, to simplify the model, no distinction is made between the source of protons involved in the reaction: both dissolved and adsorbed protons are lumped together as $H^{+}$. Hence, the rate of change of the total nitrate concentration is expressed by:

(19) $$-\frac{d}{dt}\left\{\left[NO_{3}^{-}\right]+\theta_{SHN}\;C_{TS}\right\}=k_{4}\;{\left[H^{+}\right]}^{n}\;\theta_{SHN}=k_{4}\;{\left[H^{+}\right]}^{n}\frac{K_{1}\;K_{3}\;\left[H^{+}\right]\;\left[{\mathrm{NO}}_{3}^{-}\right]}{\;1+K_{1}\left[H^{+}\right]+K_{1}\;K_{3}\;\left[H^{+}\right]\;\left[{\mathrm{NO}}_{3}^{-}\right]\;}$$

where $C_{TS}$ is the total concentration of active sites (constant) and n is the order with respect to dissolved protons of the lumped reaction. When the concentration of protons is constant, the integrated form of this equation is

(20) $$\frac{1+K_{1}\left[H^{+}\right]}{K_{1}\;K_{3}\;\left[H^{+}\right]}\ln\left(\frac{\left[{\mathrm{NO}}_{3}^{-}\right]}{{\left[{\mathrm{NO}}_{3}^{-}\right]}^{0}}\right)+\left[{\mathrm{NO}}_{3}^{-}\right]-{\left[{\mathrm{NO}}_{3}^{-}\right]}^{0}+C_{TS}\ln\left(\frac{\theta_{SHN}}{\theta_{SHN}^{0}}\right)=-k_{4}\;{\left[H^{+}\right]}^{n}\;t=-m^{\prime }\;t$$

where ${\left[{\mathrm{NO}}_{3}^{-}\right]}^{0}$ and $\theta_{SHN}^{0}$ are the corresponding equilibrium values at t=0, which can be calculated from the total amount of nitrates, dissolved or adsorbed, at t=0, $\left(C_{TN}\right)$:

(21) $$C_{TN}={\left[{\mathrm{NO}}_{3}^{-}\right]}^{0}+C_{TS}\;\frac{K_{1}\;K_{3}\;\left[H^{+}\right]\;{\left[{\mathrm{NO}}_{3}^{-}\right]}^{0}}{\;1+K_{1}\left[H^{+}\right]+K_{1}\;K_{3}\;\left[H^{+}\right]\;{\left[{\mathrm{NO}}_{3}^{-}\right]}^{0}\;}$$

**Figure 10:**
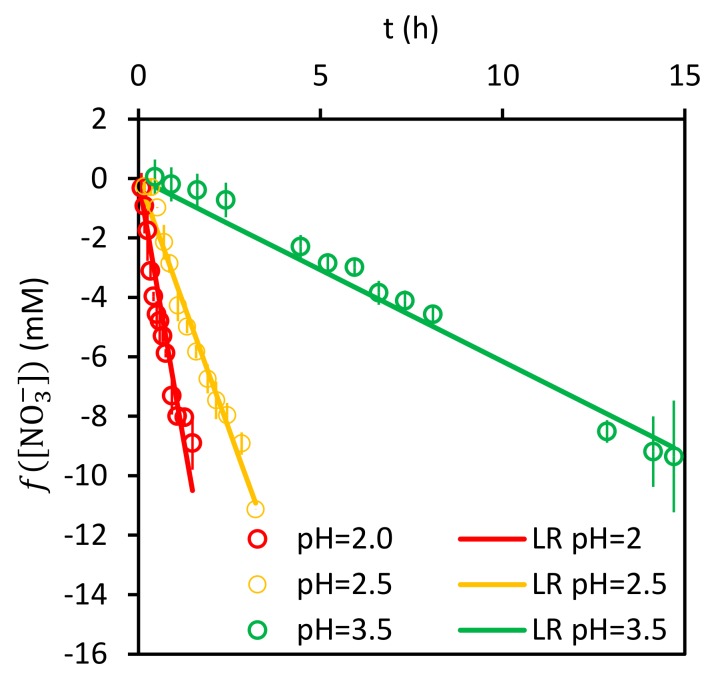
Linear regressions of experimental $f\left(\left[{\mathrm{NO}}_{3}^{-}\right]\right)$.

Let the left-hand side of the Equation (20) be $f\left(\left[{\mathrm{NO}}_{3}^{-}\right]\right)$. As long as the proton concentration remains constant, the representation of $f\left(\left[{\mathrm{NO}}_{3}^{-}\right]\right)$ vs. time should be a straight line with zero intercept. Regarding the parameters of $f\left(\left[{\mathrm{NO}}_{3}^{-}\right]\right)$, $K_{1}$ is known. Therefore, the values of $K_{3}$ and $C_{TS}$ can be obtained from a least squares simultaneous fitting of the linear regressions of the experimental data for each pH tested (Figure 10). The values obtained for these fitting parameters are: $\begin{array}{c} K_{3}=16\;200\;{\left({\mathrm{mol}\;L}^{-1}\right)}^{-1} \end{array}$ and $C_{TS}=\begin{array}{c} 0.21\;10^{-3}\;{\mathrm{mol}\;L}^{-1} \end{array}$.

**Figure 11:**
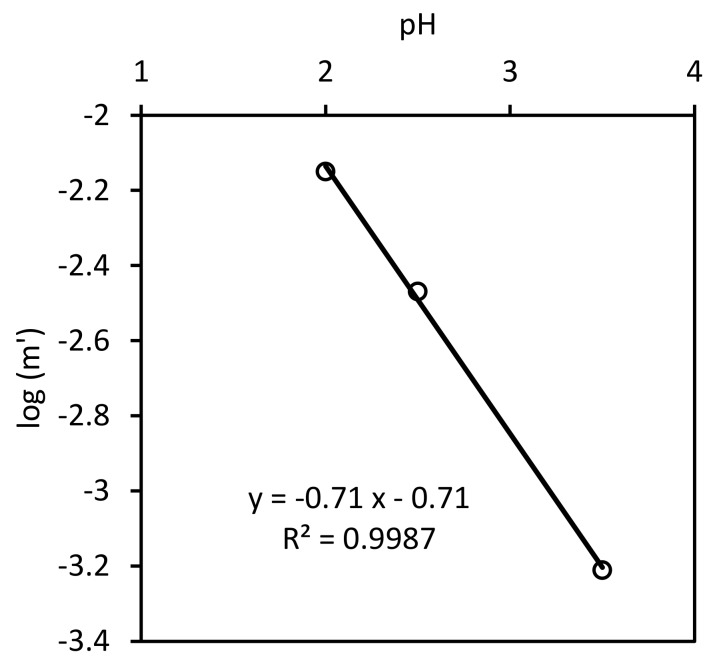
Slope values as a function of pH.

Finally, from the slopes $\left(m^{\prime }\right)$ obtained in Figure 10 for each pH value, the values of $k_{4}$ and n can be calculated; this is done after linearization of the right-hand side of Equation (20), with results presented in Figure 11. The calculated values for these parameters are: $\begin{array}{c} k_{4}=\left(0.19\pm 0.03\right) \end{array}\;h^{-1}{\left({\mathrm{mol}\;L}^{-1}\right)}^{\left(1-0.71\right)}$ and $\begin{array}{c} n=0.71\pm 0.03 \end{array}$.

**Figure 12:**
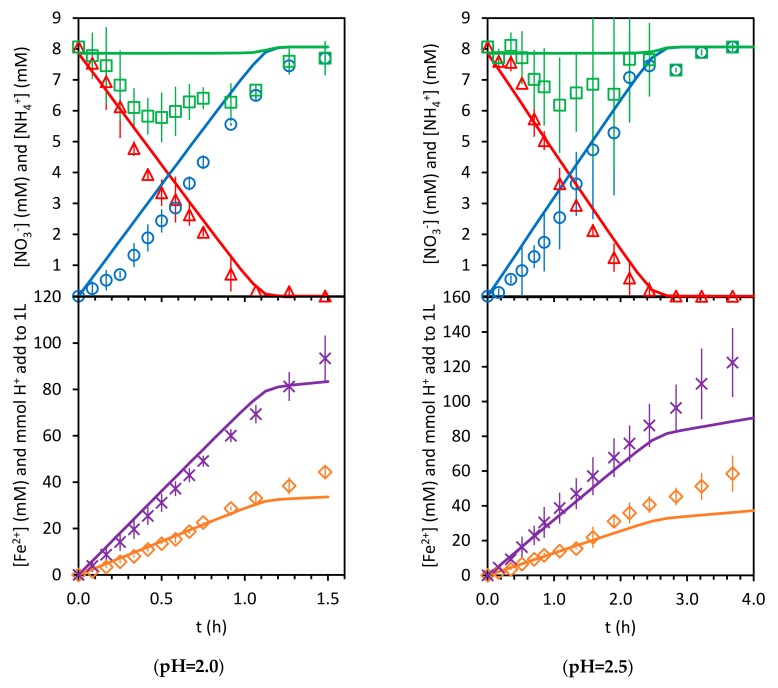
Model (solid lines) and experimental results. $△:{\mathrm{NO}}_{3}^{-}$, $○:{\mathrm{NH}}_{4}^{+}$, $□:{\left({\mathrm{NO}}_{3}^{-}+{\mathrm{NH}}_{4}^{+}\right)}_{aq}$, $\times :H^{+}$, and $◇:{\mathrm{Fe}}^{2+}$.

**Figure 13:**
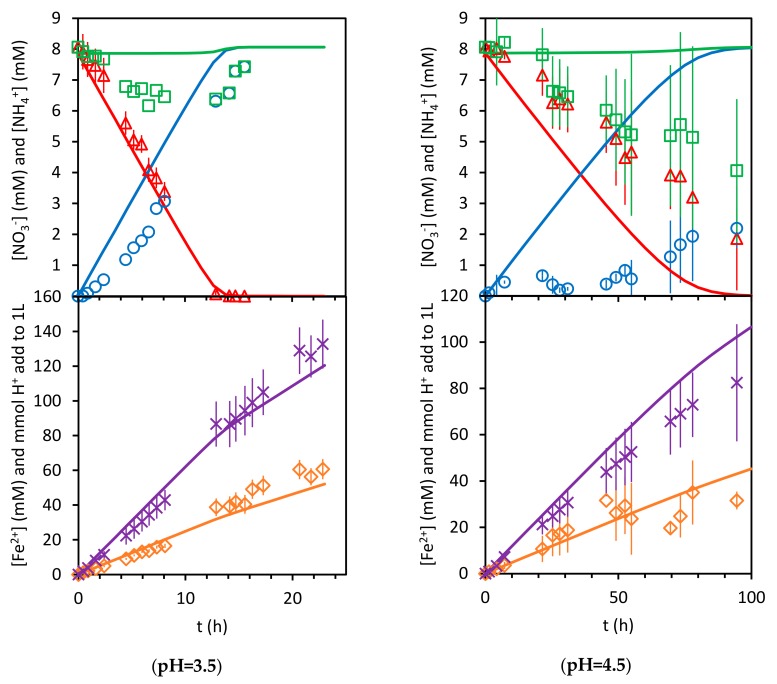
Model (solid lines) and experimental results. $△:{\mathrm{NO}}_{3}^{-}$, $○:{\mathrm{NH}}_{4}^{+}$, $□:{\left({\mathrm{NO}}_{3}^{-}+{\mathrm{NH}}_{4}^{+}\right)}_{aq}$, $\times :H^{+}$, and $◇:{\mathrm{Fe}}^{2+}$.

Figure 12 and Figure 13 present the results predicted by the model using the six fitting parameters $\left(K_{1},k_{2},\;K_{3},\;k_{4},\;n,\;\mathrm{and}\;C_{TS}\right)$ and the experimental ones. Note that the experimental results obtained at a pH value of 4.5 are shown, even when those experimental data were not used in the calculation of the model parameters values, as was explained above.

As can be seen, the model adequately reproduces the progress of nitrate concentration for the pH values of 2, 2.5, and 3.5 $\left(0.9685<R^{2}<0.9870\right)$. The sums of the aqueous concentrations of ammonium and nitrate, ${\left({\mathrm{NO}}_{3}^{-}+{\mathrm{NH}}_{4}^{+}\right)}_{aq}$, match the nitrogen balance only at the beginning and at the end of the experiments. Those differences present similar values in all the experiments. In principle, if the nitrogen species are not in the aqueous phase, they should be adsorbed in the solid phase. As can be seen, the model predicts a similar trend for the total nitrogen species in the aqueous phase, with larger values at the beginning and at the end of each experiment. Nevertheless, the calculated values for the amounts of nitrogen species under adsorption are not high enough to match the behavior of the experimental results. On the other hand, the experimental concentrations of ammonium are always below those predicted by the model, which would suggest that the mass balance differences observed for the nitrogen species probably depend more on the ammonium concentration than on the nitrate concentration. This may be indicating that the desorption kinetics of ammonium from the iron surface is not as fast as is assumed in the model.

Figure 12 and Figure 13 also show the results for ${\mathrm{Fe}}^{2+}$ concentration and acid consumption evolution. Although no model parameter was calculated from the fitting of these curves, their determination coefficient values for the results at pH values of 2 and 3.5 are between 0.9277 and 0.9756. The ones for the results at a pH value of 2.5 are not as good (0.7246 and 0.8773). This indicates a moderate reliability of the model.

## 4. Conclusions

The experimental device for the study of nitrate reduction to ammonia by the oxidation of zero valent iron (ZVI) and using a fluidized bed reactor of ${\mathrm{Fe}}^{0}$ particles allows the study of the reaction kinetics of this process at relatively low pH values, overcoming the possible mass-transfer limitations that frequently arise on relatively fast heterogeneous-reaction systems. This experimental device includes a pH control that also allows the accurate tracking of the amount of acid added to maintain the target pH value.

Under these conditions of relatively low pH values, at least the parallel reaction of $H^{+}$ reduction to $H_{2}$ should be considered, so the behavior of the system without the presence of nitrate was studied previously as blank experiments. The surface kinetic model (SKM) proposed in this manuscript allows a good prediction of the behavior of the experimental results for the blank experiments. Additionally, this kind of model is adequate for the prediction of the behavior of the experiments with nitrate reduction for pH values between 2 and 3.5. The predictions obtained for the experiments performed at pH = 4.5 are not good enough, probably due to additional reactions taking place. These satisfactory results obtained for the prediction of the system behavior were obtained with a relatively small number of fitting parameters. This small number of parameters while maintaining a good performance of the model was obtained after considering several assumptions. The main one is that the equilibrium between the dissolved and the adsorbed species before the reaction is fast enough to consider this process as being under equilibrium. 

The mass balance for the nitrogen species at the end of the experiment was quite satisfactory. Nevertheless, some discrepancies were observed for this mass balance at the intermediate stages of the nitrate reduction. One possible explanation for this could be that the desorption rate of the reduced nitrogen species is not as fast as was assumed by the model.
